# Antibody-positive pharyngeal-cervical-brachial variant superimposed on a paraparetic variant of Guillain–Barré syndrome: A case report

**DOI:** 10.1097/MD.0000000000043757

**Published:** 2025-08-15

**Authors:** Yanni Liang, Rong Fu, Jing Zhao, Zewen Chen, Chang Li, Lina Gao

**Affiliations:** a Department of Neurology, The Second People’s Hospital of Guiyang City, Guizhou, China.

**Keywords:** Guillain–Barré syndrome, paraparetic, PCB

## Abstract

**Rationale:**

Guillain–Barré syndrome (GBS) is a self-limiting immune-mediated neuropathy, pharyngeal-cervical-brachial (PCB) and paraparetic variants are rare variants of GBS spectrum disorders, and there are no reports of their coexistence.

**Patient concerns:**

The patient had an episodic course with progressive exacerbation, with frequent episodes of weakness and soreness in both lower limbs as the main complaints, and presented with dysphagia in this occurrence.

**Diagnoses:**

The patient was diagnosed with a case of GBS presenting features of both PCB and paraparetic variants.

**Interventions:**

The patient was treated with human immunoglobulin and Vitamin B1 and vitamin B12 nutritional neurotherapy.

**Outcomes:**

Owing to the clear diagnosis of GBS with features of both PCB and paraparetic variants, the patient received an intravenous infusion of 34 g of human immunoglobulin, which was received for 5 consecutive days during which the above symptoms did not occur.

**Lessons:**

A case of an anti-GT1a antibody-positive GBS variant with episodic onset is reported in this article. Rare and atypical symptoms are easy to misdiagnose clinically, thus the clinical data, diagnosis and treatment were analyzed to improve the understanding of the disease by clinicians.

## 1. Introduction

Guillain–Barré syndrome (GBS) is a self-limiting acute immune-mediated neuropathy, and with increased understanding of the disease, the concept of GBS spectrum disease has been proposed,^[1]^ of which 80% to 85% of cases are manifested as acute inflammatory demyelinating polyneuropathies,^[2]^ confirming that pharyngeal-cervical brachial (PCB) and paraplegia are rare variants of the GBS spectrum disorders. PCB is clinically characterized by dysphagia, weakness of the neck and upper extremities, decreased or absent tendon reflexes in the upper extremities, and minimal involvement of the lower extremities, which can present with symptoms of other variants.^[3]^ The core symptoms of paraplegia are weakness confined to the lower extremities, decreased or absent tendon reflexes, and hypesthesia in the lower extremities in some patients.^[4]^ A case of GBS with features of the PCB and paraparetic variants with positive anti-GT1a antibodies and seizures is reported here; this condition is easy to misdiagnose clinically because of rare and atypical symptoms. Thus, analyses of the clinical data, diagnosis and treatment of this case were performed to improve the understanding of this disease by clinicians.

## 2. Case presentation

The patient, a 36-year-old male, was admitted to the hospital on March 30, 2024 due to “episodic bilateral lower limb weakness for 20 + years, recurrent with dysphagia for 1 day“. The patient had developed, starting over 20 years before this hospital visit, bilateral lower limb weakness with soreness and discomfort after strenuous activities, mainly proximal, which he developed approximately 1 to 2 times/year, lasting approximately 1 hour. These episodes did not affect the daily life of the patient, therefore he did not seek attention for diagnosis and treatment. Patients older than 8 years-old have no obvious cause to aggravate the above symptoms, which manifest as squatting and requiring help to stand and needing to be supported by others to walk; soreness is still present 4 to 5 hours after the symptoms of fatigue are relieved. The patient visited the hospital and underwent tests including electromyography, muscle biopsy, head MRI and other examinations, which revealed no obvious abnormalities (no reports were available), and the above symptoms still recurred after relevant treatment without affecting daily life; treatment provided approximately 1 to 2 hours of relief. Finally, 1 day prior to the hospital visit reported here, the patient developed weakness of both lower limbs accompanied by soreness and discomfort in the limbs, mainly proximal accompanied by mild dysphagia, complaints of difficulty getting up and turning over, accompanied by difficulty standing, no chest restraint, no nausea or vomiting, no palpitation and chest tightness, no visual rotation, no amaurosis, no dyspnea, no incontinence and normal consciousness. Thus, he was admitted to the author’s hospital. The patient’s spirit, diet, and sleep were good, and the urine and bowel movements of the patient were normal, with no significant increase or decrease in weight. Before the illness, the patient denied the following: a history of strenuous exercise, cold, or diarrhea; a family history; and specific past medical history.

Physical examination: on admission, the blood pressure was 139/88 mm Hg and heart rate was 89 times/min, body temperature was 36.3°C, respiration rate was 18 times/min, BMI was 32, and cardiopulmonary and abdominal examinations revealed no obvious abnormalities. Neurological examination: clear, fluent in language, and providing pertinent answers. There was no abnormality in advanced cognitive function, and bilateral pupils were equally large, *d* = 3.0 mm, photosensitivity, adequate eye movements, and no nystagmus. Gross bilateral hearing was normal, with bilateral frontal lines, and nasolabial folds were symmetrical; tongue protrusion was centered, and the bilateral gag reflex was weakened, with a lowland drinking water test grade of 3. Bilateral facial and limb acupuncture sensation was symmetrical, with a bilateral iliopsoas muscle level of 4, and the remaining limb muscle had a strength grade of 5; the muscle tone was moderate, with no muscle atrophy. The bicep brachii reflex of both upper limbs was hyporeflexic, the triceps brachii and periosteal radial reflexes were abnormal, the knee reflexes and ankle reflexes of both lower limbs were abnormal, pathological signs were not elicited, and meningeal irritation signs were negative. Owing to the imperfections of the examination, VitB1 and VitB12 nutritional nerve treatment were received temporarily.

On the second day of ward rounds, the patient complained of dysphagia, weakness, and soreness in both lower limbs. The symptoms of severe neurological examination were basically recovered, and the positive signs of neurological examination revealed bilateral iliopsoas muscle grade 5, the tendon reflexes of the limbs were unchanged from before, and the relevant examinations were completed after admission: blood glucose was 6.0 mmol/L (after meals) and ECG showed sinus tachycardia and left axis deviation. There was no significant abnormality in endogenous creatinine clearance. Complete blood count showed a white blood cell count of 11.1 × 10^9^/L, a neutrophil count of 6.73 × 10^9^/L and a lymphocyte count of 3.61 × 10^9^/L. Among liver function items, alanine aminotransferase levels were 65 U/L, and renal function testing revealed uric acid levels of 481 μmol/L. Triglycerides were 3.44 mmol/L and among cardiac enzymes, creatine kinase levels were 381 U/L. Other tests, including 3 thyroid function-related antibodies + thyroglobulin, a complete set of thyroid function, erythrocyte sedimentation rate, 4 immunological items, 5 rheumatism, anticardiolipin antibody, antinuclear antibody spectrum, coagulation function, electrolytes, stool routine tests and infectious disease indicators revealed no obvious abnormalities. MRI of the brain revealed multiple lacunar infarcts of the brainstem and multiple abnormal signal shadows in the deep white matter of the brain (Fazekas class I). The embolic monitoring was negative. Transcranial Doppler flow mapping showed intracranial bilateral middle cerebral artery blood flow slowing, and 24-hour ambulatory blood pressure monitoring indicated 87 to 139/54 to 85 mm Hg with a mean blood pressure of 121/70 mm Hg. Electromyography: abnormal SEP in the extremities (suggestive of central lesions, localization?); multiple types of peripheral nerve damage (common peroneal nerve damage was severe, demyelination + axonopathy); negative repetitive electrical stimulation test. CT chest: multiple solid micronodules in both lungs, possible intrapulmonary lymph nodes, Lung-RADS grade of 2; scan for signs of mild fatty liver; the left adrenal gland was thickened, and hyperplasia was considered. There were no significant abnormalities in the serum antimyositis antibody spectrum (Table 1) and myasthenia gravis antibody (Table 2).

**Table 1:** Serum antimyositis antibody profile.

Projects	Detection method	Result	Range
Twenty six items in myositis spectrum			
Anti-HMGCR antibody, CBA method	CBA	Negative (–)	Negative (–)
Anti-cN1A antibody, CBA method	CBA	Negative (−)	Negative (−)
Anti-melanoma differentiation-related gene 5 (MDA5)	CBA	Negative (−)	Negative (−)
Anti-0J antibody IgG	Immunoblotting	Negative (−)	Negative (−)
Anti-KS antibody IgG	Immunoblotting	Negative (−)	Negative (−)
Anti-Z0 antibody IgG	Immunoblotting	Negative (−)	Negative (−)
Anti-HA antibody IgG	Immunoblotting	Negative (−)	Negative (−)
Anti-8c1-70 antibody, semiquantitative	Immunoblotting	Negative (−)	Negative (−)
Anti-PM-SCL100 antibody IgG	Immunoblotting	Negative (−)	Negative (−)
Anti-PM-sC175 antibody	Immunoblotting	Negative (−)	Negative (−)
Anti-Ku antibody IgG	Immunoblotting	Negative (−)	Negative (−)
Anti-RNA-PII antibody IgG	Immunoblotting	Negative (−)	Negative (−)
Anti-Th/To antibodies	Immunoblotting	Negative (−)	Negative (−)
Anti-fibrillarin antibody	Immunoblotting	Negative (−)	Negative (−)
Anti-NOR-90 antibody	Immunoblotting	Negative (−)	Negative (−)
Anti-Jo-1 antibody IgG	Immunoblotting	Negative (−)	Negative (−)
Anti-PL-7 antibody IgG	Immunoblotting	Negative (−)	Negative (−)
Anti-PL-12 antibody IgG	Immunoblotting	Negative (−)	Negative (−)
Anti-EJ antibody IgG	Immunoblotting	Negative (−)	Negative (−)
Anti-SRP antibody IgG	Immunoblotting	Negative (−)	Negative (−)
Anti-Mi-2	Immunoblotting	Negative (−)	Negative (−)
Anti-TIF1Y antibody IgG	Immunoblotting	Negative (−)	Negative (−)
Anti-Ro-52 antibody IgG	Immunoblotting	Negative (−)	Negative (−)
Anti-SAE1 antibody IgG	Immunoblotting	Negative (−)	Negative (−)
Anti-SAE2 antibody, western blotting	Immunoblotting	Negative (−)	Negative (−)
Anti-NXP2 antibody IgG	Immunoblotting	Negative (−)	Negative (−)

**Table 2:** Serum myasthenia gravis antibody profile.

Projects	Method	Results	Reference	Unit
Myasthenia gravis 2				
(AChR-Ab)	(RIA)	<0.01	Negative k < 0.25 Grzy Zcme 025–0.40 Positive ll 20.40	nmol/L
(MuSX)	(RIA)	<0.01	Negative ≤0.05 Positive >0.05	nmol/L
(LRP4)	CBA	Negative (−)	Negative (−)	
(Agrin-Ab)	CBA	Negative (−)	Negative (−)	–
(VGCC)	CBA	Negative (−)	Negative (−)	–
(Titin)	CBA	Negative (−)	Negative (−)	–
(RyR)	CBA	Negative (−)	Negative (−)	–
(S0X1)	CBA	Negative (−)	Negative (−)	–

On the fifth day after admission, the patient again presented with dysphagia and weakness in both lower limbs with soreness and discomfort, difficulty in getting up and turning over;no dizziness or headache, visual rotation, consciousness disorders, or fecal incontinence were reported, and these symptoms lasted for approximately 3 hours. Physical examination included blood pressure (136/80 mm Hg), and there were positive signs on neurological examination: dysphagia, lowland drinking water test grade of 3, bilateral iliopsoas muscle grade of 2, and tendon reflexes in all limbs were abnormal. Extraintestinal autoimmune peripheral neuropathy antibody was positive for the anti-GT1a antibody IgM (Table 3), leading to a clear diagnosis of immune-mediated neuropathies.

**Table 3:** Serum autoimmune peripheral neuropathy antibody profile.

Item	Detection method	Result	Reference range
Autoimmune peripheral neuropathy(25 items)			
Anti-sulfatide antibody IgG	Western blot	Negative (−)	Negative
Anti-GM1 antibody IgG	Western blot	Negative (−)	Negative
Anti-GM2 antibody IgG	Western blot	Negative (−)	Negative
Anti-GM3 antibody IgG	Western blot	Negative (−)	Negative
Anti-GM4 antibody IgG	Western blot	Negative (−)	Negative
Anti-GD1a antibody IgG	Western blot	Negative (−)	Negative
Anti-GD1b antibody IgG	Western blot	Negative (−)	Negative
Anti-GD2 antibody IgG	Western blot	Negative (−)	Negative
Anti-GD3 antibody IgG	Western blot	Negative (−)	Negative
Anti-GT1a antibody IgG	Western blot	Negative (−)	Negative
Anti-GT1b antibody IgG	Western blot	Negative (−)	Negative
Anti-GQ1b antibody IgG	Western blot	Negative (−)	Negative
Anti-sulfatide antibody IgM	Western blot	Negative (−)	Negative
Anti-GM1 antibody IgM	Western blot	Negative (−)	Negative
Anti-GM2 antibody IgM	Western blot	Negative (−)	Negative
Anti-GM3 antibody IgM	Western blot	Negative (−)	Negative
Anti-GM4 antibody IgM	Western blot	Negative (−)	Negative
Anti-GD1a antibody IgM	Western blot	Negative (−)	Negative
Anti-GD1b antibody IgM	Western blot	Negative (−)	Negative
Anti-GD2 antibody IgM	Western blot	Negative (−)	Negative
Anti-GD3 antibody IgM	Western blot	Negative (−)	Negative
Anti-GT1a antibody IgM	Western blot	Positive (+)	Negative
Anti-GT1b antibody IgM	Western blot	Negative (−)	Negative
Anti-GQ1b antibody IgM	Western blot	Negative (−)	Negative
Anti-myelin-associated glycoprotein antibody (MLAG-IgM)	Indirect immunofluorescence assay	Negative (−)	Negative (−)

Therefore, according to the patient’s weight in kilograms, 34 g of human immunoglobulin was received intravenously, and the infusion rate was 1.0 mL/minute (approximately 20 drops/minute) for 15 minutes without adverse reactions and then gradually accelerated. The fastest infusion speed did not exceed 3.0 mL/minute (approximately 60 drops/minute), for 5 consecutive days, during which time dysphagia and bilateral lower limb weakness symptoms were not present. Positive signs on a neurological examination were revealed at discharge. The bilateral iliopsoas muscle grade was 5, and the bilateral upper limb tendon reflex of the biceps was hypotrophic. The triceps and periosteal radial reflexes were abnormal, and the tendon reflexes of both lower extremities were abnormal. After 1 month, there was no difficulty in swallowing, no limb weakness and soreness, and there were no complaints of special discomfort for several months.

## 3. Discussion

The patient had an episodic course of disease, recurrent symptoms, progressive aggravation, episodic weakness and soreness of both lower limbs, with dysphagia as the main complaint. Owing to symptoms not lasting for a long time, the patient recovered on his own and the concerns of the patient were minimized. Later, because of frequent attacks affecting daily life and work, he came to our department for diagnosis and treatment. The patient was obese, and there was no obvious abnormality in his past history, the bilateral gag reflex was weakened on admission examination, the low-low drinking water test grade was 3, the bilateral iliopsoas muscle level was 4, and the biceps brachii reflex in both upper limbs was the hyporeflexic, the triceps brachii and the periosteal radial reflex were abnormal, the knee reflex in both lower limbs and ankles reflexes were abnormal. No tumor, inflammation, or infarct lesions were revealed by MRI examination, leading to suspicion of the immune-mediated neuropathies, but cases of onset in the form of seizures had not been reported. The clinical manifestations of immune-mediated peripheral neuropathy are very diverse, and the antibody profiles of these diseases are also highly variable; anti-peripheral nerve membrane surface glycolipid antibodies are closely related to autoimmune-mediated acute and chronic polyneuropathies, including antiganglioside and antiphospholipid antibodies. Anti-GT1a antibodies can be detected in PCBs, Miller Fisher syndrome, acute bulbar palsy, and acute motor axonal neuropathy. In combination with the clinical manifestations of patients without ophthalmoplegia, ataxia, hoarseness and other clinical manifestations, the possibility of Miller Fisher syndrome, acute bulbar palsy, acute motor axonal neuropathy and other diseases are not currently considered.

Cases of PCB-variants were first reported by Ropper in 1986,^[5]^ with the core symptoms of rapidly progressive oropharyngeal and cervicobrachial weakness with decreased or absent upper extremity tendon reflexes, which can be superimposed with other variants.^[3]^ There may also be patients with incomplete PCB, presenting with acute upper limb weakness, oropharyngeal paralysis, and cervical and brachial weakness. PCB-variant GBS is often preceded by a history of prodromal infection, and the pathogenesis is thought to be the invasion of organisms carrying the corresponding ganglioside antigens into the body, inducing specific autoantibodies production. Ganglioside GT1a is mainly distributed in oropharyngeal nerves and cervical brachial musculature, and its expression in the glossopharyngeal nerve and the vagus nerve is significantly stronger than that of GQ1b, so the appearance of anti-GT1a antibody manifests as oropharyngeal and cervical brachial muscle weakness. Among them, the anti-GT1a antibody was the most strongly associated with the PCB variant of GBS. Although there was no clear prodromal event in this patient, his bilateral gag reflex weakening, lowland drinking water test grade of 3, loss of bilateral upper limb tendon reflexes, no symptoms of neck muscle and upper limb muscle weakness, combined with positive serum antibodies GT1a suggested PCB as a diagnosis. However, the patient did not have cervical muscle and upper limb weakness, thus he was advised to pay close attention to these symptoms. Nevertheless, the patient’s weakness and soreness in both lower limbs, examination of both iliopsoas muscles, and loss of tendon reflexes in both lower limbs remained unexplained. Paraplegia in GBS is characterized by weakness confined to the lower extremities, decreased or absent tendon reflexes, and hypesthesia in the lower extremities in some patients.^[5]^ Therefore, paraplegic GBS was considered, and in summary, the patient was diagnosed with a case of GBS with features of pharyngeal-cervical brachial and paraparetic variants.

Both PCB and paraplegia are classified as variants of GBS, which is a self-limiting autoimmune disease. With increasing understanding of the disease, the concept of GBS spectrum disease was proposed,^[6]^ and the latest diagnostic classification was published in 2014.^[3]^ GBS is an inflammatory disease of the PNS, the most common cause of acute flaccid paralysis, with an annual incidence of approximately 0.81 to 1.89/100,000.^[7]^ GBS is more common in males than in females and although all age groups may be affected, the incidence increases with age. GBS presents primarily with rapidly developing weakness and sensory deficits in the extremities, and in some patients, facial nerve palsy, bulbar palsy, and even respiratory muscle involvement. Combined with the fact that this patient started with weakness and soreness in both lower limbs, the episodic course was inconsistent with the acute onset of GBS, which was relatively rare, and was likely to be misdiagnosed and missed. In some suspected cases of GBS, GBS variants or other uncertain cases, it is particularly important to detect serum antibody levels as soon as possible; unfortunately, the patient refused to complete the lumbar puncture.

GBS should be clinically distinguished from posterior circulation stroke, myasthenia gravis, brainstem or spinal cord inflammation or infection, mitochondrial myopathy, acute rhabdomyolysis, botulism, multiple sclerosis, and intracranial tumors.^[6]^ The patient had no history of strenuous exercise, no history of botulism, nausea or vomiting, headache, dizziness, dry mouth and other poisoning symptoms. A brain MRI was completed early and showed no imaging findings of brainstem stroke, multiple sclerosis, intracranial tumors or inflammatory lesions. The clinical manifestations also did not conform to the relapsing and remission characteristics of myasthenia gravis, which showed that the repetitive electrical stimulation test was negative, and that the above suspicious diagnoses were ruled out one by one.

Considering the patient’s response to treatment, combined with his clinical manifestations, positive anti-GT1a antibody and the results of neuroelectrophysiological examination, the diagnosis of GBS with features of PCB and paraparetic variants was confirmed. The mainstay of treatment includes intravenous immunoglobulin, plasma exchange, and pulse hormone therapy, and the prognosis is good for most patients.^[8]^ The patient had frequent seizures, was considered to be acute, had a good expected outcome after immunoglobulin infusion and has not had seizures for several months of follow-up. Therefore, early identification of clinical symptoms and timely immune intervention are essential to reduce the occurrence of severe patients, shorten the length of hospital stay, and reduce mortality.

## Author contributions

**Conceptualization:** Rong Fu.

**Formal analysis:** Jing Zhao, Chang Li.

**Funding acquisition:** Lina Gao.

**Investigation:** Zewen Chen.

**Writing – original draft:** Yanni Liang.
